# Advent of Continents: A New Hypothesis

**DOI:** 10.1038/srep33517

**Published:** 2016-09-27

**Authors:** Yoshihiko Tamura, Takeshi Sato, Toshiya Fujiwara, Shuichi Kodaira, Alexander Nichols

**Affiliations:** 1 Japan Agency for Marine-Earth Science and Technology, Yokosuka 237-0061, Japan

## Abstract

The straightforward but unexpected relationship presented here relates crustal thickness to magma type in the Izu-Ogasawara (Bonin) and Aleutian oceanic arcs. Volcanoes along the southern segment of the Izu-Ogasawara arc and the western Aleutian arc (west of Adak) are underlain by thin crust (10–20 km). In contrast those along the northern segment of the Izu-Ogasawara arc and eastern Aleutian arc are underlain by crust ~35 km thick. Interestingly, andesite magmas dominate eruptive products from the former volcanoes and mostly basaltic lavas erupt from the latter. According to the hypothesis presented here, rising mantle diapirs stall near the base of the oceanic crust at depths controlled by the thickness of the overlying crust. Where the crust is thin, melting occurs at relatively low pressures in the mantle wedge producing andesitic magmas. Where the crust is thick, melting pressures are higher and only basaltic magmas tend to be produced. The implications of this hypothesis are: (1) the rate of continental crust accumulation, which is andesitic in composition, would have been greatest soon after subduction initiated on Earth, when most crust was thin; and (2) most andesite magmas erupted on continental crust could be recycled from “primary” andesite originally produced in oceanic arcs.

The Earth is the only planet in the solar system that contains both oceanic and continental crust^1^,^2^. Without its continents, the evolution of the Earth and the life that flourishes on it would have been very different. In Earth’s early history, when continental crust was sparse, Archean oceans would have occupied a large part of its surface area^3^,^4^. Devoid of continents, Earth would have been enveloped by a monotonous crust of basalt and covered by ocean.

Continental crust formation requires juvenile input from the mantle; its evolution also involves reworking of pre-existing continental crust. When the rate of Earth’s continental crust formation is discussed, however, it is crucial to distinguish between juvenile input from the mantle and internal recycling within the crust^1^. Moreover, what is the compositional variability of the juvenile input from mantle? The continental crust we observe on the surface of the Earth has been deformed, metamorphosed, and otherwise processed perhaps several times from its creation to the present. It is impossible to imagine what wild tuna fish looks like based on what you see when you open a can of processed tuna; the same might be said about juvenile versus mature continental crust. However, we believe that the genesis of andesite in general is the key to understanding juvenile continental crust formation. The ‘andesite model’^5^ suggests that subduction zones were always the dominant sites of continent generation based on the assumption that primary arc magmas were andesitic^6^,^7^. Similarly, Kelemen *et al*.^8^ stated that ‘our favored hypothesis is that continental crust was mainly produced by fractionation of olivine and clinopyroxene from primitive andesite….interaction between eclogite melts and highly depleted mantle peridotite yielded primitive andesite’. Kushiro^9^ reviewed the experimental studies for the origin of magmas in subduction zones, those produced by partial melting of mantle peridotite under hydrous conditions, and found that melt compositions range from olivine tholeiite (basalt) to magnesian andesite. However, most subduction zone magmas, particularly those erupted along oceanic arcs, are not andesitic^10^,^11^, and it is widely concluded that granitic rocks of the continental crust are produced by partial melting of pre-existing crust (requires two stage melt generation)^12^,^13^,^14^. Currently no consensus has been reached on whether the juvenile material from the mantle wedge involved in continental crust formation is basaltic and/or andesitic. This is despite the huge number of field studies and vast amount of related analytical data that have been collected to date. However, the unexpected relationships between crustal structure and magma types presented here suggest the existence of primary (mantle-derived) andesitic magma only when the overlying crust is thin.

## Results

### Izu-Ogasawara (Bonin) –Mariana Arc

**Figure 1:**
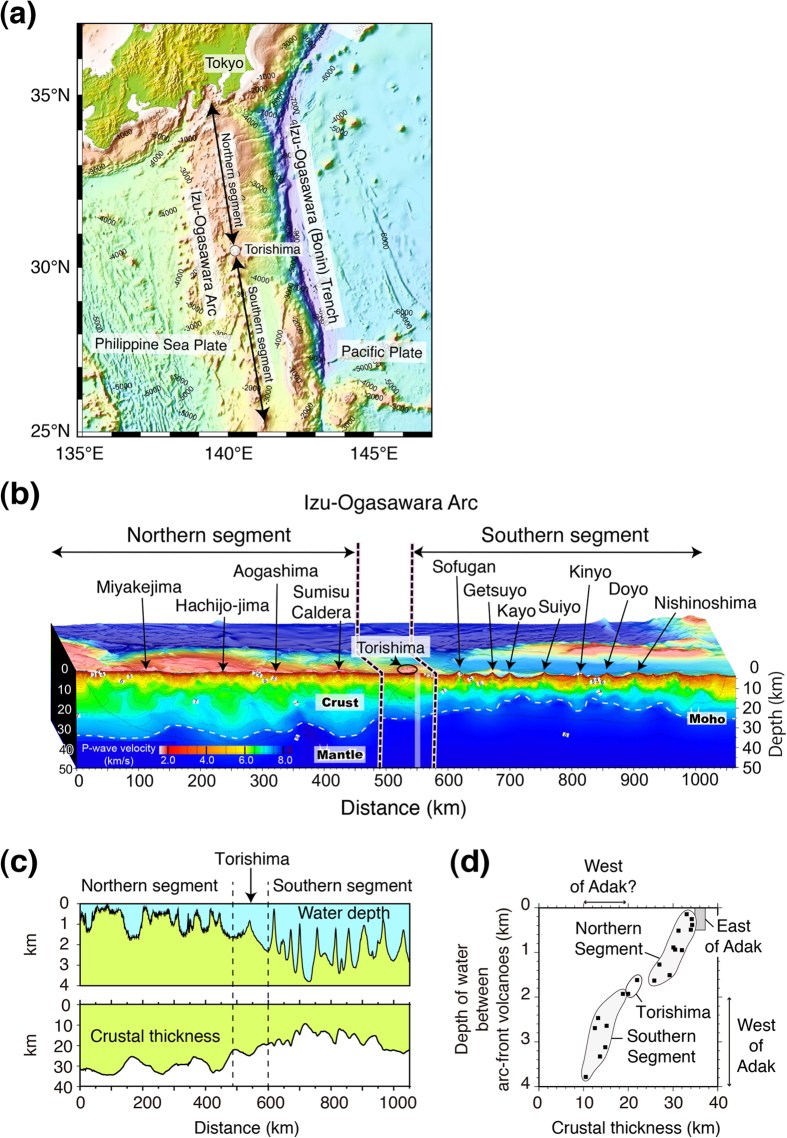
(**a**) Bathymetric features of the Izu-Ogasawara (Bonin) arcs. Old seafloor (135–180 Ma) of the western Pacific Plate subducts beneath the active Izu-Ogasawara arcs at the Izu-Ogasawara Trench. The location of the present-day volcanic front is shown by arrows, and is divided into the northern segment, the Torishima area and the southern segment, along which the wide-angle seismic profile^21^ is shown in Fig. 1b. Copyright (2007) The Geological Society of America. Reproduced with permission of GSA Publications. (**b**) Seismic velocity image along the volcanic front of the Izu-Ogasawara arcs obtained by seismic refraction tomography^21^. The crust beneath the arc volcanoes in the northern segment of the Izu-Ogasawara arcs is 32–35 km thick, which is twice as thick as the 16–21 km crust underlying the volcanoes in the southern segment. The crust beneath Torishima volcano has intermediate thickness between the northern and southern segments. Copyright (2007) The Geological Society of America. Reproduced with permission of GSA Publications. (**c**) The water depth and crustal thickness along the seismic profile, where densely deployed (~5 km spacing) ocean-bottom seismographs (OBSs) and a large air-gun array (~197 L) were used. The profile is along the volcanic front, but is just off the summits of the volcanoes. (**d**) Depth of water between arc-front volcanoes versus crustal thickness along the Izu-Ogasawara arcs. The northern segment is in shallower water and has thicker crust than the southern segment. Torishima is located and plots between them. The rectangle shows the range exhibited by the eastern Aleutian arc, east of Adak^80^.

Izu-Ogasawara-Mariana (IOM) system of arcs (also known as the Izu-Bonin-Mariana (IBM) arc system) is a typical oceanic arc produced by subduction of the Pacific Plate beneath the Philippine Sea Plate. The Izu-Ogasawara arcs extend from ~35°N near Tokyo in the north to ~24°N, the northern end of the Mariana arc in the south (Fig. 1a). Volume-weighted histograms of rock types from the northern segment of the Izu-Ogasawara arcs (30.5°–35°N), north of Torishima, are bimodal^10^, with basalt and dacite-rhyolite predominating. Rhyolitic submarine calderas^15^ are located between basalt-dominant island volcanoes, and it is suggested that rhyolite volcanoes have no mantle roots beneath the crust^16^. Instead it is believed that the rhyolite magmas are produced by melting of the existing underlying Oligocene middle crust triggered by basalt dikes (reflector X^17^) that travel laterally from the neighboring basalt volcanoes^16^. Thus, rhyolitic caldera-forming volcanoes likely resulted from crustal melting, and are omitted from discussion in this paper. The spacing of basalt-dominant island volcanoes in the northern segment is greater (~100 km) than the spacing of mostly submarine volcanoes in the southern segment (~50 km) (Fig. 1b). Supplementary Table S1 provides the sources of the analytical data from Quaternary volcanoes in the Izu-Ogasawara arcs (locations shown in Fig. 1b) used in this study. The table also shows data from Oligocene volcanic rocks collected at Omachi seamount in the Ogasawara arc^18^ and Guam, Rota and Saipan in the Mariana forearc^18^,^19^. Geochemical data from the entire IOM system of arcs from the Oligocene to the present, plus thin-section photos and videos taken from JAMSTEC submersibles, are available on the GANSEKI database^20^.

### Differing crustal thicknesses and water depth

Kodaira *et al*.^21^ conducted active source wide-angle seismic studies for ~1,050 km along the volcanic front of the Izu-Ogasawara arcs (Fig. 1a–c). These studies provide unique along-strike images of arc crust and uppermost mantle to complement earlier, cross-arc lithospheric profiles^22^ and reveal the crust of the northern segment of the Izu-Ogasawara arcs to be notably thicker than the crust of the southern segment. In the northern segment (left side of panel of Fig. 1b), the observed range of the crustal refraction phase, reflecting crustal thickness, is more than three times that of the southern section^21^. This difference indicates a considerable increase of crustal volume northward towards the Izu arc. Overall, the seismic image using these data shows that the largest volume of crust (~35 km thick) underlies the northern segment beneath Hachijo-jima, and that the smallest volume of crust (~10 km thickness) underlies the area between the Kayo and Suiyo seamounts in the southern segment, where the major tectonic line (the Sofugan tectonic line), dividing the Izu and Ogasawara arcs, crosses the volcanic front^21^. This 10-km-thick crust represents the thinnest arc-crust thickness reported anywhere on Earth^21^. Crustal thicknesses underlying each volcano in Fig. 1b are shown in Supplementary Table S1. All volcanoes along the southern segment, except for Sofugan and Nishinoshima, are submarine, and the crust underlying Nishinoshima is only 21 km thick. Nishinoshima, therefore, is one of the closest arc volcanoes to the mantle on Earth. The crust beneath Torishima volcano has a thickness of 25 km, which is intermediate between the northern and southern segments (Fig. 1b). Figure 1c shows the water depth and crustal thickness along the seismic profile, which was obtained using densely deployed (~5 km spacing) ocean-bottom seismographs (OBSs) and a large air-gun array (~197 L). The profile is along the volcanic front, but is just off the summits of volcanoes. Based on the profile, the depth of water at the spaces between arc-front volcanoes is shown versus the crustal thickness along the Izu-Ogasawara arcs in Fig. 1d. Tamura *et al*.^23^ showed that Quaternary volcanoes in NE Japan are generally underlain by topographically elevated basement rocks, which reflect ongoing uplift of the underlying basement rocks by magmatic intrusions. Thus, the water depth at the spaces between volcanoes along the volcanic front is more suitable to see the relationship between the water depth of the seafloor and crustal thickness. The northern segment is in shallower water depths and has thicker crust than the southern segment, suggesting that the depth of water over the arc massif is related to crustal thickness via isostacy. Torishima is located and plots between the two segments.

### Water depth along the Aleutians and possible crustal thickness

**Figure 2:**
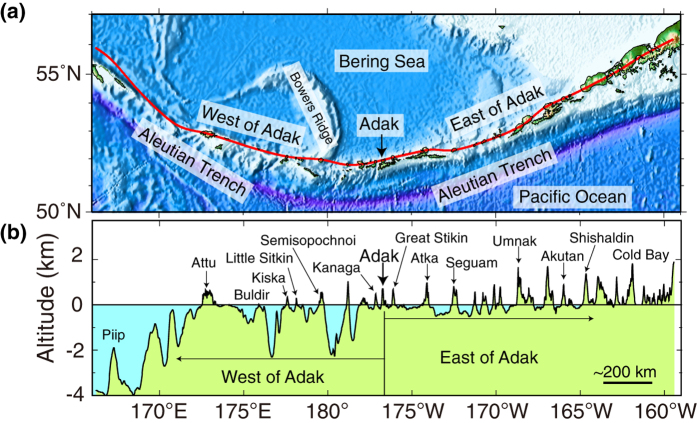
(**a**) Bathymetric features of the Aleutian arc system. The red line in the bathymetric map indicates the location of the bathymetry profile shown in Fig. 2b. The Generic Mapping Tools (GMT) software (version 4.5, https://www.soest.hawaii.edu/gmt) was used in the bathymetric data processing. Bathymetry is taken from ETOPO1^81^ (http://www.ngdc.noaa.gov/mgg/global/global.html, date of access:09/03/2016). (**b**) Bathymetry of the Aleutian arc through the arc volcanoes, from Piip submarine volcano in the west to the Pavlof volcano on the Alaska Peninsula in the east. The bathymetric data were sampled at intervals of 0.015° (~1.1 km) along the track from ETOPO1^81^ (http://www.ngdc.noaa.gov/mgg/global/global.html, date of access:09/03/2016). The water depths between volcanoes change drastically from the western Aleutian arc (west of Adak, 2,000–4,000 m) to the eastern Aleutian arc (east of Adak, 0–500 m).

Figure 2 shows the bathymetric features of the Aleutian arc system. The red line in Fig. 2a defines the bathymetry section shown in Fig. 2b, passing through the Aleutian arc volcanoes, from Piip submarine volcano in the west to Pavlof volcano on the Alaska Peninsula in the east. The water depths between volcanoes change drastically from the western Aleutians (west of Adak, 2,000–4,000 m) to the eastern Aleutians (east of Adak, 0–500 m). The range of water depths (<500 m) and crustal thicknesses (35–37 km) east of Adak are shown as a rectangle in Fig. 1d, and are consistent with the northern segment of the Izu-Ogasawara arcs. The crustal thickness west of Adak is not independently known. However, Fig. 1d suggests, based on the water depth between arc front volcanoes, that it could be similar to the thickness beneath the southern segment of the Izu-Ogasawara arcs, which is about 10–20 km thick (Fig. 2).

### Different Magma Chemistry

**Figure 3:**
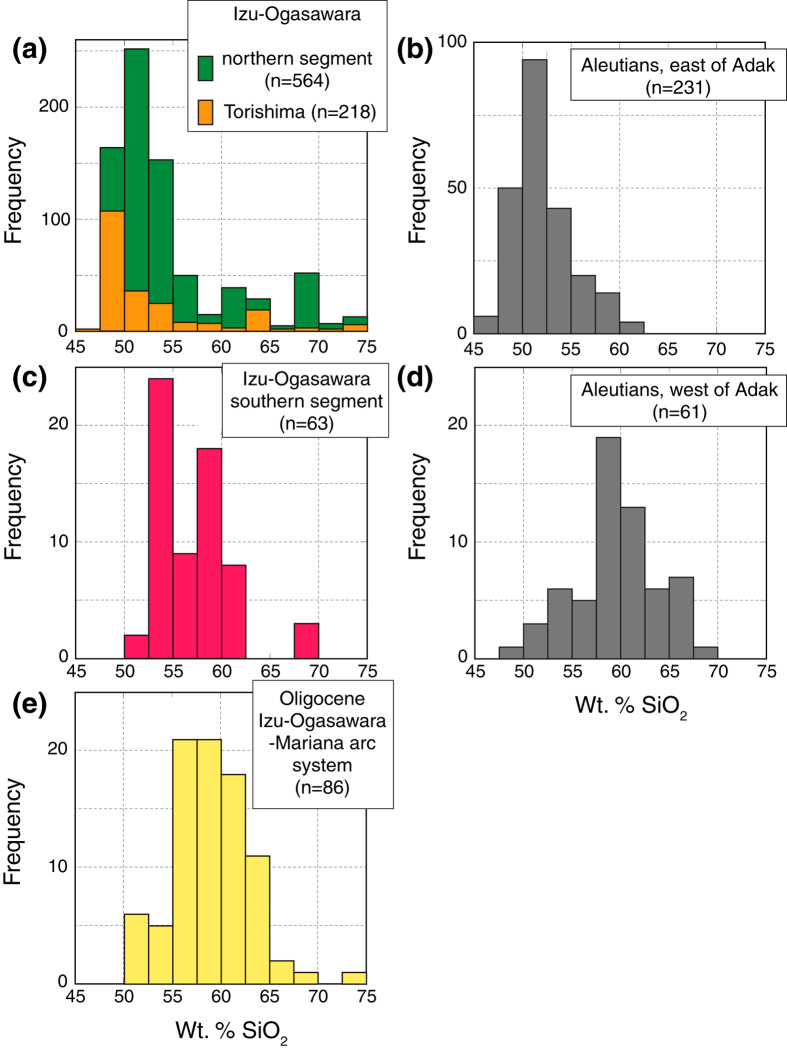
Number-of-analyses histograms of SiO_2_ content from Quaternary volcanoes along, (**a**) the northern segment of the Izu-Ogasawara arcs and Torishima, (**b**) Aleutian arc, east of Adak^24^, (**c**) the southern segment of the Izu-Ogasawara arcs, (**d**) Aleutian arc, west of Adak^24^, and (**e**) Oligocene lavas from the IOM system of arcs. Basalt lavas (<53 wt. % SiO_2_) are dominant eruptive products in the northern segment of the Izu-Ogasawara arcs, at Torishima and east of Adak, but andesites (53–63 wt. % SiO_2_) show major peaks in the southern segment of the Izu-Ogasawara arcs, west of Adak, and in the IOM arcs during the Oligocene.

Figure 3 shows histograms of SiO_2_ abundances from Quaternary volcanoes along, (a) the northern segment of the Izu-Ogasawara arcs and Torishima, (b) the Aleutian arc, east of Adak^24^, (c) the southern segment of the Izu-Ogasawara arcs, (d) the Aleutian arc, west of Adak^24^, and (e) Oligocene lavas from the IOM system of arcs. Five Quaternary volcanoes along the northern segment (Fig. 3a) of the Izu-Ogasawara arcs, eight Quaternary volcanoes along the southern segment (Fig. 3c), and Oligocene lavas from the IOM system of arcs (Fig. 3e) are based on data from references contained in Supplementary Table S1. Basalt lavas (<53 wt. % SiO_2_) are the dominant eruptive products in the northern segment of the Izu-Ogasawara arcs, Torishima and east of Adak, but andesites (53–63 wt. % SiO_2_) show major peaks in the southern segment of the Izu-Ogasawara arcs and the western Aleutian arc (west of Adak). Counter-intuitively, magmas passing through thin crust, rather than thick crust, are andesitic (Fig. 1d). Oligocene lavas from the IOM system of arcs are similar to those of the southern segment of the Izu-Ogasawara arcs and west of Adak, tending to be more SiO_2_-rich, and having unimodal peaks of andesitic compositions ranging from 55 to 65 wt. % SiO_2_ (Fig. 3e).

**Figure 4:**
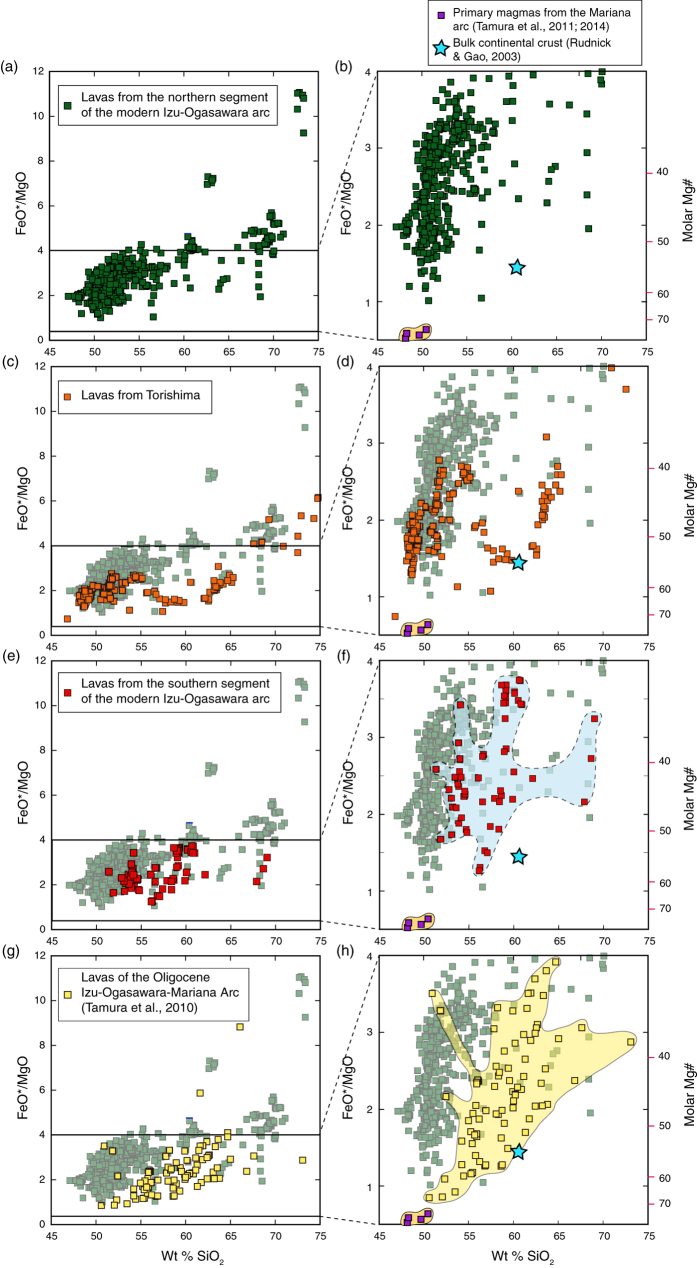
Variation diagrams of SiO_2_ (wt. %) vs FeO*/MgO ratios for lavas from (**a**,**b**) the northern segment of the present Izu-Ogasawara arcs, (**c**,**d**) Torishima, (**e**,**f**) the southern segment of the present Izu-Ogasawara arcs, and (**g**,**h**) the Oligocene Izu-Ogasawara-Mariana arcs. FeO*; total iron as FeO.

FeO*(total iron as FeO)/MgO vs. SiO_2_ diagrams show the contrast between lava compositions from the northern and southern segments of the Izu-Ogasawara arcs (Fig. 4a–f), and between the northern segment and the Oligocene IOM system of arcs (Fig. 4g,h). FeO*/MgO and the corresponding values of molar Mg# [100Mg/(Mg + Fe)] are shown on the vertical axes. Primary basaltic magmas from the Mariana arc^25^,^26^ and the bulk continental crust^27^ are also plotted. Figure 4 shows that lavas from the northern segment of the Izu-Ogasawara arcs have significantly lower SiO_2_ contents than those from the southern segment and the Oligocene IOM arcs at similar FeO*/MgO and molar Mg#. In other words, silica contents are different at the same differentiation indices reflecting crystal fractionation of olivines and pyroxenes. According to the Miyashiro definition^28^, most lavas from the northern segment are defined as tholeiitic basalts (lower SiO_2_ at a given FeO*/MgO and Mg# or molar Mg/(Mg + Fe)), whereas those from the southern segment are calc-alkaline andesites (higher SiO_2_ at the same FeO*/MgO or Mg#). Torishima, located between the segments, has features of both, containing tholeiitic and strongly calc-alkaline lavas.

The western Aleutian arc, west of Adak, has many strongly calc-alkaline andesites, including a large number of primitive andesites (Mg# >0.6)^24^,^29^. The average composition of lavas from the western Aleutian arc is the most similar to continental crust of any average oceanic arc lava composition worldwide^24^,^29^. Furthermore, isotope data from the western part of the Aleutian arc preclude recycling of components from subducted, continentally-derived sediments^24^,^29^. Thus, primitive andesites are being extracted directly from the mantle (and perhaps subducting oceanic crust) to form juvenile continental crust in the western Aleutians^24^,^29^.

### Thickness of crust and mantle melting

Mantle-derived (primary) magma stalls and accumulates in crustal magma chambers, which results in crystal fractionation of phenocryst phases and assimilation of wall rocks^30^,^31^. It seems logical, therefore, that thicker crustal sections would provide greater opportunities for magma differentiation and more evolved compositions would erupt above such crust. The unexpected relationship observed for the Izu-Ogasawara arcs and the Aleutian arc is that SiO_2_ content at a given Mg# is higher where the crust is thinner (Figs 1, 2, 3, and 4). Moreover, the immature arc lavas produced by the IOM system of arcs in the Oligocene are similarly enriched in SiO_2_ at a given Mg# compared to lavas from the northern segment of the Izu-Ogasawara arcs above thicker crust (Figs 3 and 4). The crustal thickness in the Oligocene is not really known, and subsequent rifting and extension will have thinned the crust, as represented by the southern segment of the Izu-Ogasawara arc^32^. However, we can estimate the crustal thickness in the Oligocene based on the crustal evolution of the Izu-Ogasawara arcs^33^. Kodaira *et al*.^33^ show that once steady state subduction had been established, the arc crust evolved through continuous thickening from the Eocene to the present. Thus, the Oligocene crust could have been much thinner than the crust presently underlying the northern segment of the Izu-Ogasawara arcs.

These lines of evidence indicate that the thickness of the crust in the oceanic arc influences mantle melting and the production of primary andesitic magmas rather than the extent of differentiation of similar primary basalt magmas. Thus we further explore the assumption that the thickness of the crust influences mantle melting.

### Primary magmas

Unfortunately, arc lavas are characteristically evolved, multiply-saturated, and rich in phenocrysts. As shown in Fig. 4, primitive arc magmas, representing magmas still nearly in equilibrium with mantle peridotite (FeO*/MgO <1.0 and Mg# ~70), are rare in the Izu-Ogasawara arcs. New research strategies, in particular those focusing on the lower flanks of intra-oceanic arc volcanoes using remotely operated vehicles (ROVs), are allowing us to sample magmas that may have bypassed crustal magma chambers^25^,^26^. For example the ability to collect primitive magmas from the submarine flanks of Pagan and NW Rota-1 volcanoes in the Mariana arc at ~2,000 m below sea level permitted the estimation of primary magmas compositions that are in equilibrium with the underlying mantle peridotite (Fig. 4). These primary magmas are basaltic in composition, and most differentiated basalts and basaltic andesites of the Izu arc can be explained by olivine, clinopyroxene and plagioclase fractionation from similar basaltic progenitors^34^,^35^. By using the chemical composition of primitive NW Rota-1 basalts, the primary basalt magmas were estimated to have segregated from their mantle source region at pressures of 1.5–2.0 GPa (equivalent to depths of 50–65 km)^25^. These depths are similar to the equilibration depths of 34–87 km estimated for hydrous melts beneath the Mariana volcanic arc based on thermobarometry^36^. Thus, we have not seen primary magmas that have been produced at shallower depths (<30 km) in the mantle wedge in the IOM system of arcs.

Why are basaltic rocks currently so rare in the southern segment of the Izu-Ogasawara arcs, the western Aleutians, and in the IOM arcs during the Oligocene, in spite of their thin and, in the case of the Oligocene IOM arcs, immature crust (Figs 1d and 3)? Primary magmas of calc-alkaline andesites have not yet been collected from the submarine flanks of the volcanoes in these parts of the arcs, except for some primitive andesites from the western Aleutians. However, calc-alkaline magmas and high Mg# andesites could not be produced from basaltic primary magmas through crystal fractionation, but they instead could be produced from andesitic primary magmas^8^,^37^,^38^,^39^. A shallow mantle origin for primitive andesites, and an arc origin for continental crust, seems to be consistent with data from the Aleutians^8^,^24^.

Primitive andesites are also present in the eastern Aleutian arc, most notably at Recheshnoi Volcano on Umnak Island, where the crustal thickness is known. However, the andesites from Recheshnoi volcano with Mg# of ~0.7 are from rock unit Qrq, a postglacial, quartz- and olivine-bearing, high-MgO hypersthene andesite that clearly represents a mixed magma^40^, which is different from the primitive andesites in the western Aleutians.

### Water alone is not enough to produce primary andesite

In hydrous conditions the silica content of magmas formed by partial melting of mantle peridotite increases^41^,^42^,^43^. Moreover, the presence of H_2_O increases the depth range over which tholeiite and other relatively silica-enriched magmas can be formed by partial melting of mantle peridotite^44^,^45^,^46^,^47^,^48^. Experimental melts of lherzolite KLB1 at 1.0 GPa are basaltic (SiO_2_, 50~53 wt. %) with lower H_2_O contents and become more silica-rich and high-magnesia andesitic at higher H_2_O contents^49^,^50^. Gaetani & Grove^51^ conducted detailed experiments to determine compositions of melts in equilibrium with lherzolite mineral assemblages at 1.2–2.0 GPa with up to 12 wt. % H_2_O in the melts. They showed that both SiO_2_ activity and SiO_2_ content (anhydrous base) of melts increase with greater amounts of dissolved H_2_O. However, the increase in the SiO_2_ content in partial melts is significantly less than would be necessary to produce andesitic magmas from partial melting of hydrous mantle peridotite^51^.

Melt inclusions within olivines in high Mg#, calc-alkaline andesites from near Mt. Shasta, California, USA, contain 3–6 wt. % H_2_O, not the 10–14 wt. % H_2_O that would be required to stabilize andesite in equilibrium with olivine + orthopyroxene at 1 GPa^52^. However, erupted andesites may have lost 6–8 wt. % H_2_O during mid-crustal degassing in a magma chamber, prior to entrapment of the melt inclusions, and so the inclusions may not retain the originally higher H_2_O contents of the parental magma. Such extreme H_2_O-loss from a melt (or melt inclusion), however, would induce considerable amounts of multi-phase crystallization that is not observed^52^. Theoretically, H_2_O, K_2_O and Na_2_O all displace the composition of melts in equilibrium with mantle olivine + orthopyroxene to higher SiO_2_ contents at a given pressure e.g. refs 43 and 47. However, mantle-derived primary andesites imply magma generation processes related to the presence of refractory harzburgite only in the shallow mantle (<1 GPa).

### Depths of segregation of primary magmas and their compositions

The mantle wedge in subduction zones is hydrous because of the addition of hydrous fluid from the subducting slab. Moreover, the differences of subduction components (hydrous carbonatite melt versus hydrous silicate melt) do not have a strong influence on major element compositions^25^,^26^. Thus, the effect of pressure could be the most important factor controlling mantle phase relations and the production of primary magmas with different major element compositions.

**Figure 5:**
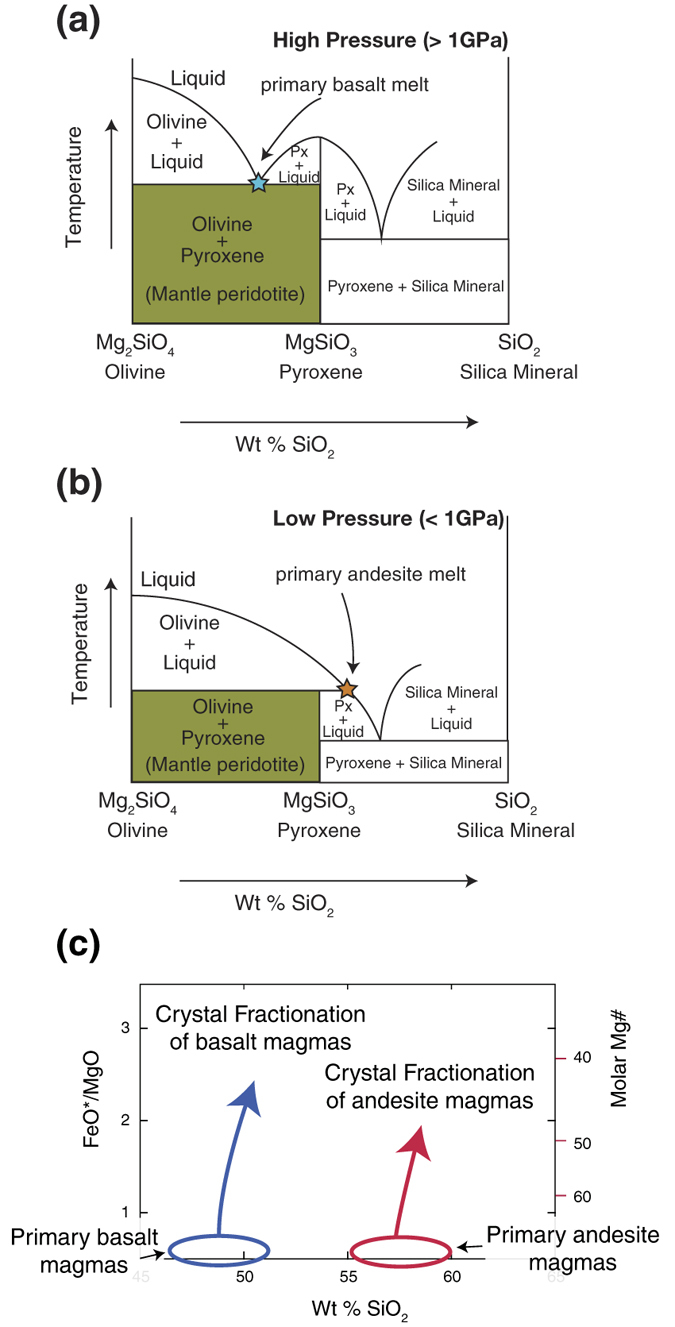
Schematic illustration of the effect of pressure on forsterite-enstatite equilibria in the hydrous environment. Primary magmas in equilibrium with magnesian olivine and magnesian orthopyroxene become progressively more silica-rich with decreasing depth^53^. (**a**) At high pressure and in hydrous conditions, congruent melting of magnesian orthopyroxene results in primary basalt melt. (**b**) At lower pressure and in hydrous conditions the liquidus field of forsterite expands relative to that of enstatite, with the result that, at some point, enstatite melts incongruently to produce primary andesite melt. (**c**) Schematic illustration showing primary basalt and andesite magmas and their possible fractionation trends.

Figure 5 shows the schematic effect of pressure on forsterite-enstatite equilibria in hydrous conditions. Similar diagrams can be seen in many petrology textbooks, based on high-pressure experiments under dry conditions^53^. Primary magmas in equilibrium with magnesian olivine and orthopyroxene become progressively more silica-rich or silica-poor with decreasing and increasing depths, respectively. A) At high pressure (>~1 GPa) and in hydrous conditions, congruent melting of magnesian orthopyroxene results in only primary basalt melt. B) At lower pressure (<~1 GPa) and in hydrous conditions, the liquidus field of forsterite expands relative to that of enstatite with the consequence that at some point, enstatite melts incongruently to produce primary andesite melt.

The schematic illustration in Fig. 5c shows primary basalt and andesite magmas and their possible fractionation trends. Most basalts and basaltic andesites of the northern segment of the Izu-Ogasawara arcs and the eastern Aleutian arc could be produced from primary basalt magmas through crystal fractionation. On the other hand, basaltic lavas are rare in the southern segment of the Izu-Ogasawara arcs and western Aleutian arc where the crust is thin, and in the IOM arc system during the Oligocene (Fig. 3) when the crust was thin. It is possible that the calc-alkaline andesites or high Mg# andesites in these arcs were produced by crystal fractionation of primary andesite magmas, not primary basalt magmas.

There may be regions in the upper mantle beneath arcs that are not composed of peridotite, but instead have been transformed to pyroxenite via reaction with relatively SiO_2_-rich magmas. The SiO_2_-rich magma reactants may form either during cooling and crystal fractionation in the shallow mantle^54^,^55^ or by melting of subducting basalt and sediment, followed by transport in diapirs to the shallow mantle, together with reaction between the melt and mantle at high melt/rock ratios^55^,^56^,^57^. Based on the phase diagrams, however, modal volume change of the source mantle via reaction with relatively SiO_2_-rich magmas does not change our conclusions that olivine exists in the source mantle (Fig. 5a,b). Although some areas of the mantle wedge of subduction zones could be completely transformed to pyroxenite in which there are no olivines, such extreme transformation while ubiquitous will be minimal in volume.

### Crustal thicknesses control the stall-depths of mantle diapirs and thus the composition of primary magmas

**Figure 6:**
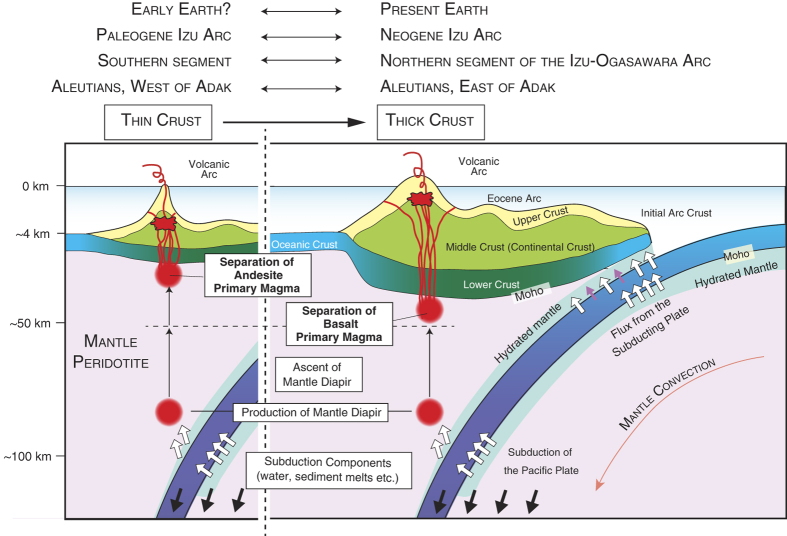
Along-arc variation of crustal thicknesses along the Izu-Ogasawara arcs. Partial melting of mantle wedge can result in the production of mantle diapirs, which ascend into the mantle wedge. Where the crust is thin, these diapirs could rise to shallower depths than where the crust is thick. The pressure where primary magmas separate from mantle peridotite would therefore be low where the crust is thin and higher where the crust is thick. In the former scenario andesite primary magmas are produced through incongruent melting of magnesian pyroxene; in the latter primary basalt magmas are produced through congruent melting of the same starting material. The Early Earth, the IOM arcs during the Oligocene, the western Aleutian, west of Adak, could have been similar to the southern segment of the Izu-Ogasawara arcs.

Where the crust is thin, mantle diapirs can ascend to shallower depths below the crust and segregate primary magmas (Fig. 6). Magmas in equilibrium with mantle peridotite at low pressure (<~1 GPa) and in hydrous conditions are andesitic in composition (Fig. 5b) and can readily evolve into continental crust type (calc-alkaline andesite) magmas (Fig. 5c). In contrast, where the crust is thick, mantle diapir ascent is attenuated at greater depths and primary magmas segregate at higher pressures (Fig. 6). Under increased pressure (>~1 GPa), the liquidus field of forsterite shrinks relative to that of pyroxene (Fig. 5a) even under hydrous conditions. Thus the eutectic melt becomes poorer in silica content. All magmas in equilibrium with mantle peridotite at higher pressures could be basaltic in composition, as we have estimated for the Mariana arc^25^,^26^; these magmas cannot evolve directly into calc-alkaline andesite (continental crust) (Fig. 5c). The hypothesis proposed here, that primitive andesites originated from shallow mantle melting and continental crust formed in an arc setting, is consistent with data from the Aleutians^24^.

However, the thin crust does not result in the melting of the shallow mantle beneath the crust when the temperature is below its solidus. Moreover, basaltic magmas could be produced at higher pressures within the mantle wedge even when the overlying crust was thin. In short, a lack of primitive andesites or the presence of basaltic magmas from volcanoes on thin crust does not necessarily contradict the hypothesis presented here. We, however, expect that volcanoes on the thicker crust will tend to produce more basaltic magmas and cannot produce primitive andesites. This is consistent with the observations from the northern segment of the Izu-Ogasawara arc and east of Adak in the Aleutian arc (Figs 3 and 4).

### Implications for andesite genesis in continental arcs

The petrological features of andesite and dacite lavas erupted from Daisen volcano in SW Japan during the last million years can be attributed to ‘anti-fractionation’, in which episodes of heating (and remelting) of solidified andesite protolith produced the compositional variations observed in the volcanic rocks at the surface^58^. This model proposes that andesite and dacite eruptions were triggered by the influx of hot basalt magmas from the mantle, which reheated, softened and reactivated crustal andesite magma bodies at depth, permitting them to erupt. Reheating and remobilization of calc-alkaline magmas has been envisaged in the Adamello massif, Italy^59^, Lascar volcano, Chile^60^, and Soufriere Hills volcano, Montserrat^61^,^62^,^63^. Price *et al*.^64^ present a model for the generation of Ruapehu andesitic magmas in New Zealand and the evolution of crust in that continental subduction setting. This example demonstrates that, in continental andesite volcanoes, whole-rock compositions are not necessarily direct analogues for melt compositions^65^,^66^,^67^,^68^,^69^,^70^,^71^. Segregation of partial melt from restite crystals produces magma of rhyolitic composition. Partial melting of previously emplaced, intermediate calc-alkaline rocks can produce the chemical compositions of the silicic group of the voluminous Tiribi Tuff (~25 km^3^) in Central Costa Rica^72^. The Izu arc is characterized by bimodal, basalt**-**rhyolite, magmatism, and rhyolite could be produced by dehydration melting of solidified hydrous calc-alkaline andesite in the arc crust^10^,^16^. Yogodzinski & Kelemen^73^,^74^ show that strongly calc-alkaline magmas in the Aleutians, with evidence for magma mixing, incorporate a primitive mantle-derived andesite or dacite endmember.

Most andesitic and silicic magmas erupted on continental crust and thick oceanic crust (>30 km) could therefore be recycled from “primary” andesite previously produced in oceanic arcs when the crust was thin.

### Implications for continental crust formation

Plate tectonics processes may have initiated at about 3.8 Ga^1^,^75^,^76^, and the volume of continental crust may have progressively increased from that early time. This is very controversial, however, as can be seen from inspection of Fig. 1 of Korenaga^76^. Stern and Scholl^77^ refute the arguments that continental crust volume has increased with time since plate tectonics began. The southern segment of the Izu-Ogasawara arcs and the western Aleutian arc where the crust is ~10–20 km thick, however, could be an analogue for an early stage subduction zone. Bulk arc crust is different from bulk continental crust, as suggested by many authors^14^,^21^,^78^. However, arc-arc collision such as at the Tanzawa mountains in Japan, delaminates lower crust from the upper and middle crust, resulting in andesitic bulk composition^18^,^79^. Thus, subduction involving thin crust produces andesite, which is the material of continental crust, and subsequent arc-arc collisions accumulate these materials to produce continents. It is therefore suggested that a “stockpile” of continental crust (andesitic magma) was produced when most crust was thin.

However, it should be noted that it has taken about 50 and 40 million years for the Izu-Ogasawara arcs and the Aleutian arc, respectively, to attain their current thicknesses. The thicker, Kohistan arc crust exposed in northern Pakistan formed in 50 to 70 million years. These time spans are small compared to the duration of the Archean and Proterozoic eras. There was plenty of time to form thick arc crust in the early Earth. On the other hand, it could be argued that, in a hotter early Earth, the extents of melting were higher in mantle plumes, beneath spreading ridges, and in arcs. If so, perhaps the crust in such settings was generally thicker, rather than thinner, compared to the present day. Thus, the production of continental crust might have been impossible in the early Earth if the oceanic crust had been thick.

## Discussion

The relationship between crustal thickness and magma compositions in the Izu-Ogasawara arcs and the Aleutian arc suggests that shallow mantle melting in hydrous conditions is crucial for the genesis of andesitic magmas. Moreover, andesite is produced only when the crust is thin, thus only in oceanic arcs. If so, most andesite magmas erupted on continental crust could be recycled from “primary” andesite previously produced in oceanic arcs. A “stockpile” of continental crust (andesitic magma) might be produced when most crust was thin. Although the crustal thickness in the early Earth is quite controversial, the rate of continental crust accumulation might have been greatest early in Earth’s history when subduction was initiated and if the initial crust was thin. On the other hand, if the crust was generally thicker, rather than thinner, compared to the present day, the production of continental crust might have been impossible in the early Earth.

## Additional Information

**How to cite this article**: Tamura, Y. *et al*. Advent of Continents: A New Hypothesis. *Sci. Rep.* **6**, 33517; doi: 10.1038/srep33517 (2016).

## Supplementary Material

Supplementary Information
