# Supplementary material for: Advent of Continents: A New Hypothesis
Source: Sci Rep. 2016 Sep 27;6:33517. doi: 10.1038/srep33517 (PMC5037404; doi:10.1038/srep33517)
Supplement: Supplementary Information [file srep33517-s1.pdf]

Advent of Continents. A New Hypothesis

Yoshihiko Tamura*, Takeshi Sato, Toshiya Fujiwara, Shuichi Kodaira and Alexander Nichols
Japan Agency for Marine-Earth Science and Technology, Yokosuka 237-0061, Japan
Telephone: +81-46-867-9761. Tax: +81-46-867-9625.
E-mail: tamuray@jamstec.go.jp

Supplementary Table S1 Sources of analytical data of the Quaternary volcanoes of the Izu-Ogasawara (Bonin) Arc and lavas from the Oligocene Izu-Ogasawara-Mariana Arc

Volcano (number of analyses)	Latitude	Crustal thickness[21] (km)	References
**Northern segment of the Izu-Ogasawara Arc**			
Miyakejima (135)	34°05'	35	1, 2, 3, 4, 5, 6, 7, 8
Hachijo-jima (199)	33°08'	35	9, 10, 11, 12
Aogashima (106)	32°28'	32	13, 14
Sumisu Caldera (97)	31°26'	35	15
Torishima (217)	30°29'	25	14, 16
**Southern segment of the Izu-Ogasawara Arc**			
Sofugan (4)	29°47'	21	17
Getsuyo (9)	29°18'	21	17, 18
Kayo (3)	29°04'	16	17, 18
Suiyo (3)	28°35'	17	17, 19
Mokuyo (9)	28°20'	?	17, 19
Kinyo (17)	28°05'	21	17, 20, 21
Doyo (3)	27°40'	17	17, 19
Nishinoshima (16)	27°15'	21	18, 22
**Oligocene Arc**			
Omachi Seamount			23
Guam, Rota and Saipan			23, 24

Published and unpublished geochemical data, some photos of thin sections and existing videos took by JAMSTEC submersibles (Shinkai 2000, Shinkai 6500

and Hyper-Dolphin 3000) are available on the GANSEKI database[20].

1. Aramaki, S. & Hayakawa, Y. The October 1983 eruption of Miyakejima volcano. *Sci. Resul. Monbusho* (No. 58022004), 13-31 (in Japanese) (1984).
2. Fujii, T. Aramaki, S., Fukuoka, T. & Chiba, T. Petrology of ejecta and lavas of the 1983 eruption of Miyake-jima. *Bull. Volcanol. Soc. Japan* **29,** S266-S282 (in Japanese with English abstract and figure captions) (1984).
3. Soya, T. et al. Bulk and mineral chemistry of lavas and ejecta of the 1983 eruption of Miyakejima volcano. *Bull. Volcanol. Soc. Japan* **29,** S283-S296 (in Japanese with English abstract and figure captions) (1984).
4. Aramaki, S., Hayakawa, Y., Fujii, T., Nakamura, K. & Fukuoka, T. The October 1983 eruption of Miyakejima volcano. *J. Volcanol. Geotherm. Res.* **29,** 203-229 (1986).
5. Sato, J., Nakamura, T., Takahashi, H. & Sato, K. Elemental compositions of recent eruptive products from Miyake-jima volcano. *Bull. Volcanol. Soc. Japan* **41,** 269-274 (in Japanese with English figure captions) (1996).
6. Amma-Miyasaka, M. & Nakagawa, M. Recent magma plumbing system beneath Miyake-jima volcano, Izu islands, inferred from petrological study of the 1940 and 1962 ejecta. *Bull. Volcanol. Soc. Japan* **43,** 433-455 (in Japanese with English abstract and figure captions) (1998).
7. Yokoyama, T., Kobayashi, K., Kuritani, T. & Nakamura, E. Mantle metasomatism and rapid ascent of slab components beneath island arcs: Evidence from ^{238}U-^{230}Th-^{226}Ra disequilibria of Miyakejima volcano, Izu arc, Japan. *J. Geophys. Res.* **108**, doi:10.1029/2002JB002103 (2003).
8. Yokoyama, T., Kuritani, T., Kobayashi, K. & Nakamura, E. Geochemical evolution of a shallow magma plumbing system during the last 500 years, Miyakejima volcano, Japan: Constraints from ^{238}U-^{230}Th-^{226} Ra systematics. *Geochim. Cosmochim. Acta* **70,** 2885-2901 (2006).
9. Tsukui, M., Suzuki, M. & Sano, A. Evolution of magma plumbing system of Hachijo-Higashiyama volcano in the last 30000 years. *Bull. Volcanol. Soc. Japan* **38,** 199-212 (in Japanese with English abstract and figure captions) (1993).

10. Nakano, S., Yamamoto, T. & Isshiki, N. Chemical compositions of the Hachijojima volcano group. Izu Islands: comparison between the Higashiyama and Nishiyama volcanoes based on chemical analyses of surficial samples. *Bull. Geol. Surv. Japan* **48,** 93-105 (in Japanese with English abstract and figure captions) (1997).
11. Hirata, D., Yamashita, H. & Taniguchi, H. Geochemical composition of the core sample (N2-HJ-4) in Hachijojima. In: *Report of the 1996-1998 Tokutei-B of Earthquake Research Institute, University of Tokyo* (ed Nakada, S.) 27-34 (Tokyo University, 1999).
12. Ishizuka, O., Geshi, N., Itoh, J., Kawanabe, Y. & TuZino, T. The magmatic plumbing of the submarine Hachijo NW volcanic chain, Hachijojima, Japan: long-distance magma transport? *J. Geophys. Res.* **113,** doi:10.1029/2007JB005325 (2008).
13. Takada, A. et al. Geology of Aogashima volcano, Izu Islands, Japan. *Bull. Volcanol. Soc. Japan* **37,** 233-250 (1992).
14. Tokyo-To (Committee for Disaster Prevention). *Data for Studies on the Characteristics of Volcanic Eruptions in the Izu Islands Area, the Booklet of Earth Science.* (Tokyo-To, 1992).
15. Tamura, Y. et al. Are arc basalts dry, wet, or both? Evidence from the Sumisu caldera volcano, Izu-Bonin arc, Japan. *J. Petrol.* **46,** 1769-1803 (2005).
16. Tamura, Y. et al. Wet and dry basalt magma evolution at Torishima volcano, Izu-Bonin arc, Japan: the possible role of phengite in the downgoing slab. *J. Petrol.* **48,** 1999-2031. (2007).
17. Yuasa, M. & Nohara, M. Petrographic and geochemical along-arc variations of volcanic rocks on the volcanic front of the Izu-Ogasawara (Bonin) arc. *Bull. Geol. Surv. Japan* **43,** 421-456 (1992).
18. Ishizuka, O. et al. Processes controlling along-arc isotopic variation of the southern Izu-Bonin arc. *Geochem. Geophys. Geosyst.* **8,** Q06008, doi:10.1029/2006GC001475 (2007).
19. Nagaoka, S., Kasuga, S. & Kato, Y. Geology of Mokuyo Seamount, Doyo Seamount, and Suiyo Seamount in the Sitiyo Seamounts on the Ogasawara arc. *Proc. JAMSTEC Symp. Deep-Sea Res.* **8,** 237-248 (In Japanese with

English abstract) (1992).
20. Watanabe, K. & Shibata, A. Submarine volcanic topography and geology of Kinyo Seamount on the Izu-Ogasawara Arc. *JAMSTEC J. Deep Sea Res.* **12,** 239-246 (in Japanese with English abstract) (1996).
21. JAMSTEC. *Deep Seafloor Rock Sample Database (GANSEKI).* http://www.godac.jamstec.go.jp/ganseki/e, (2010) (Date of access: 22/11/2014).
22. Geological Survey of Japan, AIST. Nishinoshima volcano. https://gbank.gsj.jp/volcano/Act_Vol/nishinoshima/index.html, (2013) (Data of access: 04/07/2014).
23. Tamura, Y. et al. Missing Oligocene crust of the Izu-Bonin arc: consumed or rejuvenated during collision? *J. Petrol.* **51,** 823-846 (2010).
24. Reagan, M. K. et al. Petrogenesis of volcanic rocks from Saipan and Rota, Mariana Islands, and implications for the evolution of nascent island arcs. *J. Petrol.* **49,** 441-464 (2008).
